# Insuffisance cardiaque à fraction d'ejection preservee en afrique sub-saharienne: à propos de 32 cas

**DOI:** 10.11604/pamj.2013.16.100.2323

**Published:** 2013-11-16

**Authors:** Mouhamed Cherif Mboup, Khadidiatou Dia, Pape Diadie Fall

**Affiliations:** 1 Cardiology department, Principal Hospital of Dakar, Senegal

**Keywords:** Insuffisance cardiaque, dysfonction diastolique, Afrique sub-saharienne, heart failure, diastolic dysfunction, Sub-Saharan Africa

## Abstract

L'insuffisance cardiaque à fraction d’éjection préservée est une affection de plus en plus fréquente dont le diagnostic est difficile car elle prédomine chez les sujets âgés présentant d'autres pathologies. Le but de ce travait était d’évaluer les aspects épidémiologiques, cliniques, paracliniques et thérapeutiques de cette affection en milieu hospitalier. Il s'agit d'une étude prospective, descriptive réalisé au niveau du service de cardiologie de l'hôpital principal de Dakar sur une période de 01 an (01 février 2008 - 31 janvier 2009). Le diagnostic d'insuffisance cardiaque avait été retenu chez 111 patients. Au sein de ce groupe de patient, l’échodoppler cardiaque mettait en évidence une dysfonction diastolique isolée du ventricule gauche dans 32 cas (28,8%). L’âge moyen des patients était de 65,7 ± 9,1 ans. On notait une prédominance féminine avec sex ratio à 1,28 (18 femmes / 14 hommes). Une hypertension artérielle était retrouvée chez tous les patients. L’électrocardiogramme inscrivait une hypertrophie ventriculaire gauche et une fibrillation auriculaire chez respectivement 21 (65%) 4 patients (13%). L’échodoppler cardiaque trans-thoracique mettait en évidence une hypertrophie ventriculaire gauche et une dilatation de l'oreillette gauche chez respectivement 18 (56%) et 24 patients (75%). Le flux mitral était pseudonormal, restrictif, ou de type trouble de la relaxation chez respectivement 13 (41%), 10 (31%) et 9 (28%) patients. L'insuffisance cardiaque à fraction d’éjection préservée est caractérisée dans nos régions par la précocité de sa survenue et la présence chez tous les patients d'un long passé d'hypertension artérielle non équilibrée.

## Introduction

L'insuffisance cardiaque est un problème majeur de santé publique. L'insuffisance cardiaque à fraction d’éjection préservée (ICFEP) est une forme fréquente dont le diagnostic est difficile car elle prédomine chez les sujets âgés présentant d'autres pathologies [1]. Chez ces patients, la limitation fonctionnelle est souvent au premier plan et résume généralement l'histoire clinique à des épisodes d'insuffisance cardiaque aiguë alternant avec des périodes de latence clinique [2, 3]. Les résultats des études récentes montrent que le pronostic de l'ICFEP est mauvais avec une survie annuelle proche de celle de l'insuffisance cardiaque par dysfonction systolique du ventricule gauche [4]. Malgré ces données, la prise en charge thérapeutique de l'ICFEP n'est pas bien codifiée. C'est dans ce contexte que nous avons réalisé en Afrique sub-saharienne et plus particulièrement au Sénégal ce travail afin d’évaluer les aspects épidémiologiques, cliniques, paracliniques et thérapeutiques de cette affection en milieu hospitalier.

## Méthodes

Il s'agit d'une étude prospective, descriptive réalisé au niveau du service de cardiologie de l'hôpital principal de Dakar au Sénégal sur une période de 01 an (01 février 2008 - 31 janvier 2009). Les critères d'inclusion étaient: la présence de symptômes et/ou de signes d'insuffisance cardiaque; la mise en évidence à l’échocardiographie d'une fraction d’éjection ventriculaire gauche supérieure à 50%, et d'une dysfonction diastolique. Les paramètres échocardiographiques ont été obtenus à l'aide d'un TOSHIBA XARIO disposant d'une sonde adulte de 2,5 MHz et d'une fonction doppler tissulaire.

Le diagnostic d'une dysfonction diastolique reposait sur les recommandations de la société européenne de cardiologie. Une dysfonction diastolique était retenue devant un rapport entre la vélocité maximale du flux protodiastolique transmitral de remplissage du ventricule gauche en doppler pulsé (Em) et celle recueillie en doppler pulsé tissulaire au niveau de l'anneau mitral en protodiastole (Ea) supérieur à 15. Lorsque ce rapport était compris entre 8 et 15, le diagnostic reposait sur la mise en évidence d'un des signes suivants: une différence entre la durée du flux veineux pulmonaire contemporaine de la systole auriculaire (Ap) et celle du flux télédiastolique transmitral de remplissage ventriculaire gauche (Am) supérieure à 30 millisecondes; un temps de décélération de l'onde Em (TD) supérieur à 280 millisecondes associé à un rapport Em/Am inférieur à 0,5; une masse ventriculaire gauche supérieure à 125g/m^2^chez la femme et 149 chez l'homme; une fibrillation auriculaire à l’électrocardiogramme. Les critères d'exclusion étaient une fraction d’éjection ventriculaire gauche inférieure à 50%, une péricardite, et un rétrécissement mitral.

Les paramètres étudiés étaient: les éléments épidémiologiques: la prévalence, l’âge, le sexe, les antécédents; les aspects cliniques; les examens paracliniques: la biologie, l’électrocardiogramme, l’échocardiographie Doppler; les modalités de prise en charge, et l’évolution intra-hospitalière. Les paramètres étudiés avaient été consignés sur une fiche d'enquête informatisée à l'aide du logiciel Epi info version 6.0 de l'Organisation Mondiale de la Santé. L'analyse des données avait été réalisée avec le logiciel SPSS 11.0 (Statiscal Package For Social Sciences).

## Résultats

Durant la période concernée par l’étude 587 patients avaient été hospitalisés dans le service. Le diagnostic d'insuffisance cardiaque avait été retenu chez 111 patients soit une prévalence hospitalière de 19%. Au sein de ce groupe de patient, l’échodoppler cardiaque mettait en évidence une dysfonction diastolique isolée du ventricule gauche dans 32 cas. (prévalence de 28,8%).

### Caractéristiques cliniques

L’âge moyen des patients était de 65,7 ± 9,1 ans. On notait une prédominance féminine avec sex ratio à 1,28 (18 femmes / 14 hommes). La recherche de facteurs de risque cardio-vasculaire mettait en évidence une hypertension artérielle chez tous les patients avec une durée moyenne d’évolution de 9,25 ± 3,5 ans. Un diabète de type II et un tabagisme actif et actuel étaient retrouvés chez respectivement 13 (45%) et 6 patients (9%). Une obésité était retrouvée chez 16 patients (50%) avec un body mass index (BMI) moyen de 25,55 ± 4,52 kg/m^2^. Un antécédent d'hospitalisation pour insuffisance cardiaque était présent chez 10 malades (31%). Deux patients (6%) avaient été hospitalisés dans le service pour un infarctus du myocarde. La symptomatologie était dominée par la dyspnée d'effort présente chez 27 patients (84%). Elle pouvait être isolée ou associée à une orthopnée ou à une dyspnée paroxystique nocturne chez respectivement 13 (41%) et 6 (19%) patients. L'examen physique mettait en évidence des signes d'insuffisance cardiaque congestive chez 13 patients (41%). Les données de l'examen clinique sont résumées au niveau du Tableau 1.

**Tableau 1:** Données de l'examen clinique

Signes et Symptômes	Patients	%
Dyspnée d'effort	27	84%
Dyspnée de repos	13	41%
Dyspnée paroxystique nocturne	6	19%
Toux	13	41%
Œdèmes des membres inférieurs	13	41%
Tachycardie	14	43%
Bruit de galop	4	13%
Râles crépitants	14	44%
Râles sibilants	2	6%
Hépatomégalie	9	28%
Turgescences des veines jugulaires	6	19%
Reflux Hépato-jugulaire	6	19%

### Caractéristiques Paracliniques

L’électrocardiogramme inscrivait une hypertrophie ventriculaire gauche et une fibrillation auriculaire chez respectivement 21 (65%) 4 patients (13%). Chez 2 patients (6,25%) on notait des séquelles de nécrose en antéro-septal.

Sur le plan biologique, une anémie était présente chez 15 patients (47%). La clairance de la créatinine était de 55,48 ± 35,35 ml/mn avec un taux sérique de la créatinine à 21,59 ± 22,40 mg/l. Une altération de la fonction rénale était retrouvée chez 26 patients (72%). L'insuffisance rénale était terminale chez 4 malades (12,5%), modérée et sévère chez respectivement 17 (53%) et 2 (6%) patients. Le taux moyen de cholestérol total était de 2,05 ± 0,77 g/l. Cependant, une hypercholestérolémie n’était présente que chez 12 malades (37,5%).

L’échodoppler cardiaque trans-thoracique mettait en évidence une hypertrophie ventriculaire gauche chez 18 patients (56%) avec une masse à 114,40 ± 33,37 mg/m^2^. L'oreillette gauche était dilatée chez 30 patients (94%) avec une surface à 25,07 ± 6,78 cm^2^. Le flux mitral était pseudonormal, restrictif, ou de type trouble de la relaxation chez respectivement 13 (41%), 10 (31%) et 9 (28%) patients. Un rapport Em/Ea supérieur à 15 était présent chez 23 patients (72%) permettant de retenir le diagnostic d'insuffisance cardiaque à fonction systolique préservée.

Chez les 9 (28%) autres patients, ce diagnostic était retenu devant une différence Ap-Am supérieure à 30 ms chez 5 (16%) malades, une masse ventriculaire gauche supérieure à 125g/m^2^ chez 2 (6%) patientes et une fibrillation auriculaire sur l’électrocardiogramme dans 2 (6%) cas. Une hypertension artérielle pulmonaire était retrouvée chez 11 (34%) patients avec une pression artérielle pulmonaire systolique à 53,95 ± 15,04 mmHg comme le montre le Tableau 2.

**Tableau 2:** Paramètres échocardiographies

Paramètres	Moyennes	Déviations
Diamètre télédiastolique du ventricule gauche (mm)	48,88	7,57
Diamètre télésystolique du ventricule gauche (mm)	30,05	7,54
Fraction d’éjection du ventricule gauche	67,02	11,23
Masse ventriculaire gauche indexée (g/m^2^)	114,40	33,37
Volume oreillette gauche indexée (ml/m^2^)	52,5	3,53
Surface de l'oreillette gauche (cm^2^)	25,07	6,78
Diamètre de l'oreillette gauche (mm)	42,69	5,67
Flux protodiastolique trans-mitral Em (cm/s)	91,38	31
Flux télédiastolique trans-mitral Em Am (cm/s)	71,98	25,62
Temps de décélération (ms)	204,53	57,57
Onde (Ea) au Doppler tissulaire (cm/s)	5,99	2,83
Ratio Em/Ea	16,67	5,57
Différence Ap-Am (ms)	38,20	7,18
Pression artérielle pulmonaire systolique (mmHg)	53,95	15,04

### Traitement et evolution

Le traitement médical de l'insuffisance cardiaque à fraction d’éjection préservée est représenté dans le Tableau 3 avec une place prépondérante au furosémide utilisé chez tous nos patients. En association au furosémide, les diurétiques thiazidiques et la spirinolactone ont été utilisés chez respectivement 4 (13%) et 3 (9%) malades. Les inhibiteurs de l'enzyme de conversion (IEC) ont été prescrits chez 26 (81%) patients. En cas d'intolérance au IEC, les antagonistes des récepteurs AT1 de l'angiotensine II étaient utilisés chez 5 (16%) patients. En association au inhibiteur du système rénine angiotensine aldostérone, les inhibiteurs calciques et les bêtabloquants ont été prescrits chez respectivement 16 (50%) et 13 (41%) patients.

**Tableau 3:** Traitement

Traitements	Patients	%
Furosemide	32	100%
Thiazidique	4	13%
Spirinolactone	3	9%
Inhibiteur de l'enzyme de conversion	26	81%
Antagoniste des récepteur de l'angiotensine II	5	16%
Inhibiteur Calcique	16	50%
Bêtabloquant	13	41%
Anti-vitamine K	3	9%
Aspirine	5	16%
Antidiabétique oral/ Insuline	9	28%

L’évolution a été favorable chez tous nos malades avec une durée d'hospitalisation 10,32 ± 5,6 jours.

## Discussion

L'insuffisance cardiaque constitue une affection de plus en plus fréquente en Afrique sub-saharienne, où on assiste à une transition épidémiologique caractérisée par l’émergence des affections non transmissibles. Dans notre travail, 28,8% des patients hospitalisés pour une insuffisance cardiaque présentaient une dysfonction diastolique isolée. Cette prévalence de l'ICFEP se rapproche de celle retrouvée dans des études américaines similaires 24 à 55% [6–8]. Dans le travail de Kingue et al au Cameroun [9], cette prévalence était de 10%. Cependant dans cette étude certains paramètres de la dysfonction diastolique tel que le doppler tissulaire ou le flux veineux pulmonaire n'avaient pas été appréciés et pourraient rendre compte d'une sous estimation de la fréquence de cette pathologie en Afrique sub-saharienne.

Notre étude confirme la prédominance féminine de l'ICFEP avec un sex-ratio à 1,28. Cependant l’âge moyen de nos patients (65,7 ± 9,1 ans) est inférieur à ceux retrouvés dans les séries européennes et nord américaines. Dans ces études, l’âge moyen était de 78 ± 10 ans et 75,1 ± 13,1 ans respectivement en France et aux états unis d'Amérique [10, 11]. Cette disparité pourrait être liée d'une part à l'espérance de vie plus faible en Afrique sub-saharienne mais surtout à l'inaccessibilité de la majorité de la population aux structures sanitaires permettant un dépistage précoce et une prise en charge efficiente des différents facteurs de risque cardio-vasculaire concourant à la survenue de cette affection. Ces facteurs de risque cardio-vasculaire sont dominés par l'hypertension artérielle présente chez tous nos malades. L'hypertension artérielle constitue le facteur de risque cardio-vasculaire le plus fréquemment rencontré chez les patients présentant une ICFEP [12, 13]. Dans le travail de Nurcan et al chez les Afro-Américains, l'hypertension artérielle était retrouvée chez 96.1% des patients présentant une dysfonction diastolique. La longue évolution (durée moyenne d’évolution de 9,25 ± 3,5 ans) d'une hypertension artérielle non équilibrée aboutit à la survenue d'une cardiopathie hypertensive. Elle se caractérise par l'apparition d'une hypertrophie ventriculaire gauche avec une augmentation de la rigidité artérielle et ventriculaire, et une altération de la relaxation [12]. La prévalence du diabète dans notre travail (45%) s'apparente à celle (42.3%) retrouvée dans le travail de Nurcan et al chez les Afro-Américains [13]. Dans l’étude de Tribouilly, le diabète constituait un facteur de mauvais pronostic chez les patients hospitalisés pour une ICFEP. Sa présence était associée à une survie à 05 ans de 32% et une augmentation de la mortalité de 60% par rapport au groupe de patients non diabétiques [14].

Chez nos patients, l'ICFEP était associée à deux autres comorbidités: l'insuffisance rénale et l'anémie. L'insuffisance rénale résulte chez ces malades d'une nephroangiosclérose, en rapport avec une hypertension artérielle ancienne (durée moyenne d’évolution de 9,25 ± 3,5 ans) et non équilibrée. L'insuffisance rénale sévère chez ces patients, multiplie le risque de complications cardio-vasculaires par le biais de modifications structurelles du myocarde, de l'anémie et d'une intolérance aux excès de sel [10, 15]. La prévalence de l'anémie dans notre travail (47%) s'apparente à celle retrouvée dans l’étude de Shamagian et al (46%) [16]. La présence d'une anémie dans ce travail était significativement associée à d'autres facteurs de mauvais pronostique tel que l'insuffisance rénale, et la cardiopathie ischémique. Elle constituait en analyse multivariée un facteur prédictif indépendant de mortalité, multipliant le risque de décès par 2.7 [16].

L'hypertrophie ventriculaire gauche et la dilatation de l'oreillette gauche constituent les deux anomalies morphologiques échocardiographiques les plus fréquemment retrouvées chez nos patients et dans la littérature [17].

La dilatation de l'oreillette gauche retrouvée chez 94% de nos patients, témoigne de la chronicité et de la sévérité de la dysfonction diastolique du ventricule gauche [18, 19]. Elle favorise la survenue d'une fibrillation auriculaire présente chez 13% de nos patients. La prévalence de la fibrillation auriculaire chez les patients hospitalisés pour une ICFEP est importante aussi bien dans les études épidémiologiques (30% à 40% [20, 21]) et dans les études randomisées (20% à 30% [22–24]). Dans le travail de Fung et al, la fibrillation auriculaire (29%) était associée à une classe fonctionnelle NYHA élevée, à une altération du test de marche de 6 minutes, et à une dilatation de l'oreillette gauche [25]. Elle constituait un facteur pronostique péjoratif indépendant dans l’étude CHARM (Candesartan in Heart Failure) [26]. La tachycardie, la perte de la systole auriculaire, et l'irrégularité des cycles cardiaques constituent autant de mécanismes pouvant expliquer l'impact péjoratif de la fibrillation auriculaire chez les patients présentant une insuffisance cardiaque à fraction d’éjection préservée [25].

## Conclusion

L'insuffisance cardiaque à fraction d’éjection préservée constitue une affection de plus en plus rencontrée en pratique quotidienne en Afrique sub-saharienne. Elle se caractérise dans nos régions par la précocité de sa survenue et l'existence chez tous les patients d'un long passé d'hypertension artérielle non équilibrée. C'est dire tout l'intérêt du dépistage et de la prise charge de l'hypertension artérielle qui doivent être intégrés dans un programme global de lutte contre les maladies cardio-vasculaires. C'est une affection grave du fait des nombreuses comorbidités qui lui sont associées, justifiant la réalisation d'autres travaux pour évaluer son pronostic à long terme dans nos régions.

## References

[1] Pérez de Isla L, Cañadas V, Contreras L (2009). Diastolic heart failure in the elderly: In-hospital and long-term outcome after the first episode. Int J Cardiol..

[2] Cohen Solal A (2002). Diastolic heart failure: myth or reality?. Eur J Heart Fail..

[3] Yamasaki N, Kitaoka H, Matsumura Y, Furuno T, Nishinaga M, Doi Y (2003). Heart failure in the elderly. Intern Med..

[4] Bhatia SR, Jack TV, Lee DS (2006). Outcome of Heart Failure with Preserved Ejection Fraction in a Population-Based Study. N Engl J Med..

[5] Paulus WJ, Tscho C, Sanderson EJ (2007). How to diagnose diastolic heart failure: a consensus statement on the diagnosis of heart failure with normal left ventricular ejection fraction by the Heart Failure and Echocardiography Associations of the European Society of Cardiology. Eur Heart J..

[6] Malki Q, Sharma ND, Afzal A (2002). Clinical presentation, hospital length of stay, and readmission rate in patients with heart failure with preserved and decreased left ventricular systolic function. Clin Cardiol..

[7] Philbin EF, McCullough PA, Dec GW (2001). Length of stay and procedure utilization are the major determinants of hospital charges for heart failure. Clin Cardiol..

[8] Vaccarino V, Gahbauer E, Kasl SV (2002). Differences between African Americans and whites in the outcome of heart failure: evidence for a greater functional decline in African Americans. Am Heart J..

[9] Kingue S, Dzudie A, Menanga A, Akono M, Ouankou M, Muna W (2005). Nouveau regard sur l'insuffisance cardiaque chronique de l'adulte en Afrique à l’ère de l’échocardiographie Doppler: expérience du service de médecine de l'Hôpital Général de Yaoundé. Annal Cardiol Angéiol..

[10] Roux E, Pieri B, Bergeri I, Jauffret B, Villeneuve L, Arquès S (2003). Facteurs aggravants associés à l'insuffisance cardiaque à fonction systolique conservée chez le sujet âgé. Ann Cardiol Angeiol..

[11] Fonarow GC, Stough WG, Abraham WT (2007). Characteristics, Treatments, and Outcomes of Patients With Preserved Systolic Function Hospitalized for Heart Failure A Report From the OPTIMIZE-HF Registry. JACC..

[12] Diamond JA, Phillips RA (2005). Hypertensive heart disease. Hypertens Res..

[13] Nurcan I, Hoffman M, Moore RH, Easley K, Jacobson TA (2006). Comparison of African-American Patients With Systolic Heart Failure Versus Preserved Ejection Fraction. Am J Cardiol..

[14] Tribouilloy C, Rusinaru D, Mahjoub H (2008). Prognostic impact of diabetes mellitus in patients with heart failure and preserved ejection fraction: a prospective five-year study. Heart..

[15] Fatema K, Hirono O, Takeishi Y (2002). Hemodialysis improves myocardial interstitial edema and left ventricular diastolic function in patients with end-stage renal disease: noninvasive assessment by ultrasonic tissue characterization. Heart vessels..

[16] Shamagian G, Roman AV, Garcia-Acun MJ, Ramos PM, Lamela AV, Gonzalez-Juanatey JR (2006). Anaemia is associated with higher mortality among patients with heart failure with preserved systolic function. Heart..

[17] Berry C, Hogg K, Norrie J, Stevenson K, Brett M, McMurra J (2005). Heart failure with preserved left ventricular systolic function: a hospital cohort study. Heart..

[18] Danzmann LC, Bodanese LC, Köhler I, Torres MR (2008). Left atrioventricular remodeling in the assessment of the left ventricle diastolic function in patients with heart failure: a review of the currently studied echocardiographic variables. Cardiovasc Ultrasound..

[19] Maeder MT, Kaye DM (2009). Heart Failure With Normal Left Ventricular Ejection Fraction. J Am Coll Cardiol..

[20] Owan TE, Hodge DO, Herges RM, Jacobsen SJ, Roger LV, Redfield MM (2006). Trends in prevalence and outcome of heart failure with preserved ejection fraction. N Engl J Med..

[21] Hogg K, Swedberg K, McMurray J (2004). Heart failure with preserved left ventricular systolic function; epidemiology, clinical characteristics, and prognosis. J Am Coll Cardiol..

[22] McMurray JJ, Carson PE, Komajda M (2008). Heart failure with preserved ejection fraction: clinical characteristics of 4133 patients enrolled in the I-PRESERVE trial. Eur J Heart Fail..

[23] Yusuf S, Pfeffer MA, Swedberg K (2003). Effects of candesartan in patients with chronic heart failure and preserved left-ventricular ejection fraction: the CHARM-Preserved trial. Lancet..

[24] Cleland GJ, Tendera M, Adamus J, Freemantle N, Polonski L, Taylor J (2006). The perindopril in elderly people with chronic heart failure (PEP-CHF) study. Eur Heart J..

[25] Fung WJ, Sanderson EJ, Yip GW, Zhang Q, Yu CM (2007). Impact of atrial fibrillation in heart failure with normal ejection fraction: a clinical and echocardiographic study. J Card Fail..

[26] Olsson LG, Swedberg K, Ducharme A (2006). Atrial fibrillation and risk of clinical events in chronic heart failure with and without left ventricular systolic dysfunction: results from the Candesartan in Heart failure-Assessment of Reduction in Mortality and morbidity (CHARM) program. J Am Coll Cardiol..

